# Is excessive smartphone use associated with weight status and self-rated health among youth? A smart platform study

**DOI:** 10.1186/s12889-023-15037-8

**Published:** 2023-02-03

**Authors:** Kayla Brodersen, Nour Hammami, Tarun Reddy Katapally

**Affiliations:** 1 Johnson Shoyama Graduate School of Public Policy, University of Regina, 2155 College Ave, Regina, Saskatchewan S4M 0A1 Canada; 2 Trent University Durham, 55 Thornton Road South, Oshawa, Ontario L1J 5Y1 Canada; 3 DEPtH Lab, School of Health Studies, Faculty of Health Sciences, Western University, 1151 Richmond St, London, Ontario N6A 3K7 Canada; 4 Department of Epidemiology and Biostatistics, Schulich School of Medicine and Dentistry, Western University, London, Ontario N6A 3K7 Canada; 5 Children’s Health Research Institute, Lawson Health Research Institute, London, Ontario N6C2R5 Canada

**Keywords:** Digital health, Smartphone use, Weight status, Self-rated health, Youth health

## Abstract

**Background:**

In Canada, it is recommended that youth limit screen time to less than two hours per day, yet, the majority of youth are reportedly spending a significantly higher amount of time in front of a screen. This is particularly concerning given that these recommendations do not take into account smartphone devices, which is the most common screen time technology of choice for the younger generations. This study implements an innovative approach to understanding screen time behavior and aims to investigate the unique relationship between smartphone specific screen time and physical health outcomes.

**Methods:**

This cross-sectional study is part of the Smart Platform, a digital epidemiological and citizen science initiative. 436 youth citizen scientists, aged 13–21 years, provided all data via their own smartphones using a custom-built smartphone application. Participants completed a 124-item baseline questionnaire which included validated self-report surveys adapted to collect data specifically on smartphone use (internet use, gaming, and texting), demographic characteristics, and physical health outcomes such as weight status and self-rated health. Binary regression models determined the relationship between smartphone use and physical health outcomes.

**Results:**

Overall participants reported excessive smartphone use in all categories. 11.4% and 12% of the 436 youth participants reported using their smartphone excessively (greater than 2 h per day) during the week and weekend respectively for gaming and were over 2 times more likely than their peers to fall within an overweight/obese BMI status. Excessive weekend gaming was also associated with self-rated health where participants were over 2 times more likely than their peers to report poor self-rated health.

**Conclusions:**

The results indicate that excessive screen time on smartphones does have complex associations with youth health. Further investigation with more robust study designs is needed to inform smartphone-specific screen time guidelines for youth.

## Background

Populations across the world have access to digital devices and engage in screen time activities starting from early childhood [1]. While many screen time behaviors could be considered a necessary part of functioning in today’s increasingly digital society, it is important to consider relationships between excessive screen time use and population health given the abundance of evidence available that suggests there are associations between screen time behaviors and negative mental, physical, emotional, and social health outcomes [2–6]. For example, Grimaldi-Puyana, et al. (2020) show that higher levels of smartphone use are associated with more sedentary behavior time, as well as, poorer mood, sleep quality, and physical activity.

Smartphone-specific screen time is important to understand because worldwide there are over 6 billion smartphone subscriptions, and it is projected that this will grow to over 7.5 billion by 2026 [8]. In Canada, there are approximately 31.38 million smartphone users and this is projected to be over 34 million by 2024 [9]. With the high rate of smartphone market penetration [9] across the globe, it is critical to understand the relationship between smartphone use and population health.

Youth in particular are a group of special interest since they may be accessing smartphone technology even more so than any other device. Some studies in the United States show that while only 88% of teenagers report having access to a desktop or laptop computer, 95% report having access to a smartphone [10]. Smartphone use, particularly among youth, may have unique implications beyond screen time accumulated on other, non-portable digital devices. For example, this may be because youth have higher usage with smartphones than of other non-portable digital devices but also because smartphone usage goes beyond social media, texting, and internet surfing, to include video streaming and video gaming behaviours that are traditionally associated with non-portable digital devices.

In Canada, it is recommended that youth engage in less than two hours of screen time per day [11], which currently does not address smartphone use and screen time accumulated on smartphones [12]. Moreover, emerging evidence on smartphone use suggests that higher levels of smartphone use is also associated with a greater decrease in physical activity [13], sedentary behaviors [14], and higher weight status and BMI [15] within the youth population.

Current research indicates that screen time behavior will have unique associations with self-rated health. For example, studies looking at screen time behaviors, such as television viewing and computer/video gaming, showed that higher television viewing time, not computer/video gaming screen time was associated with poor self-rated health, but only for males [16]. By contrast, higher television viewing time and computer/video gaming based screen time had other negative health impacts for females; namely, with social friendships [16]. Although associations have been typically found with traditional screen time devices (i.e. television, desktop/laptop, video gaming consoles), further research is needed to investigate the relationship between smartphone-specific screen time and self-rated health. This exploratory study aimed to address key gaps in evidence by investigating the association between smartphone use (texting, internet use, gaming, and total use) and physical health (weight status and self-rated health) among youth in an urban setting in Canada.

## Methods

### Study design, recruitment, and data collection

**Fig. 1:**
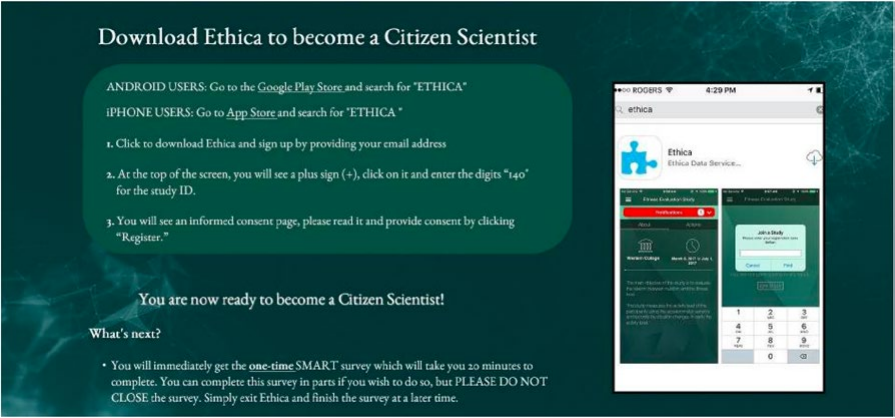
Instructions to become a citizen scientist

The data collected for this study are part of the Smart Platform, a citizen science and digital epidemiological initiative for ethical population health surveillance, integrated knowledge translation, and real-time behavioral interventions [17]. A custom-built smartphone app, developed as part of the Smart Platform, was used to engage participants as citizen scientists to capture behavior patterns and their relationship with physical health outcomes [17]. Following the instructions outlined in Fig. 1, participants had the option to download the app using both Android and iPhone smartphones through either the Google Play Store or Apple App Store [17]. Participants provided all data via their smartphones, including demographic and subjective data through survey questions using their smartphone. Previous studies have shown that smartphone apps can be used to collect valid and reliable health data in both rural and urban centres, and within diverse populations; for example, from university students and lower-income families [18]. The Smart Platform is an innovative approach that leverages the market buy-in of digital technology such as citizen-owned smartphones to better understand population health behaviors [19].

### Participants

A total of 808 youth and young adult citizen scientists (13 to 21 years) were recruited through Regina Public and Catholic School engagement sessions held in various high schools throughout Regina, Saskatchewan in 2018. All high schools in the city of Regina were approached to participate in the study. Of the 5 schools that agreed, the participation rate was over 88% of high school students (grades 9–12) across all participating schools. This indicates that the sample was representative of the youth population in an urban jurisdiction in Canada, particularly because the schools that participated belonged to neighbourhoods with varied socioeconomic status. The recruitment strategy required the development and maintenance of a collaborative relationship with school administrators to schedule in-person recruitment sessions. During each session, research team members spent time describing the study, answering questions, and assisting youth to download the study app on their smartphones.

**Fig. 2:**
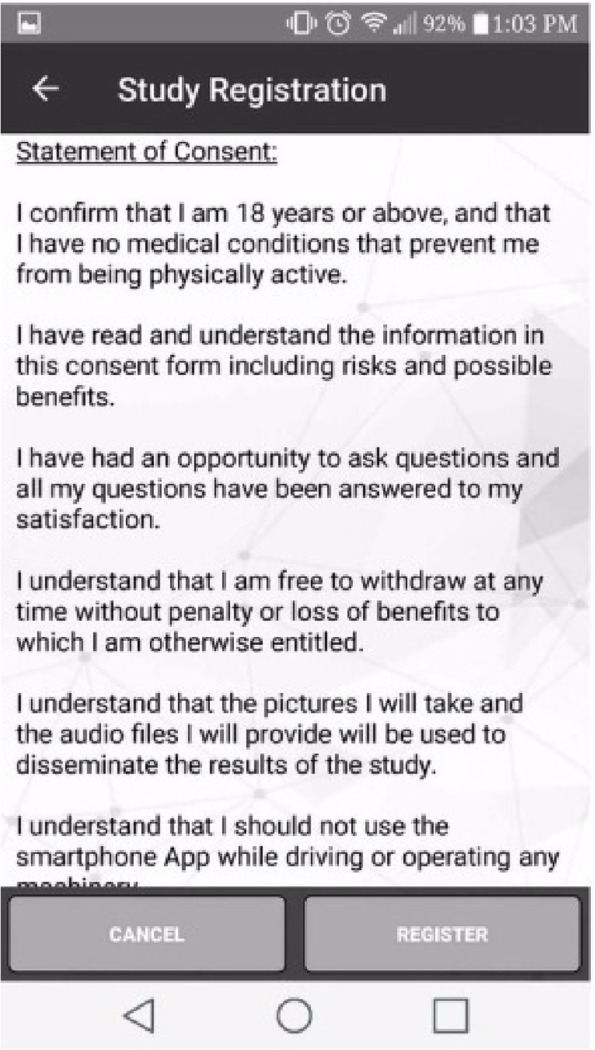
Informed Consent Form

All participants were required to confirm their age, but only the participants aged 18 years or older were required to complete informed consent (Fig. 2) via the app on their smartphone. For participants aged 13–17 years, implied informed consent was provided by parents or guardians of each youth ahead of the recruitment session so that they had the opportunity to read about the study and ask questions. If any parent or guardian did not want their child to participate in the study, they could email the team at smart.study@uregina.ca or smart.study@usask.ca.

**Fig. 3:**
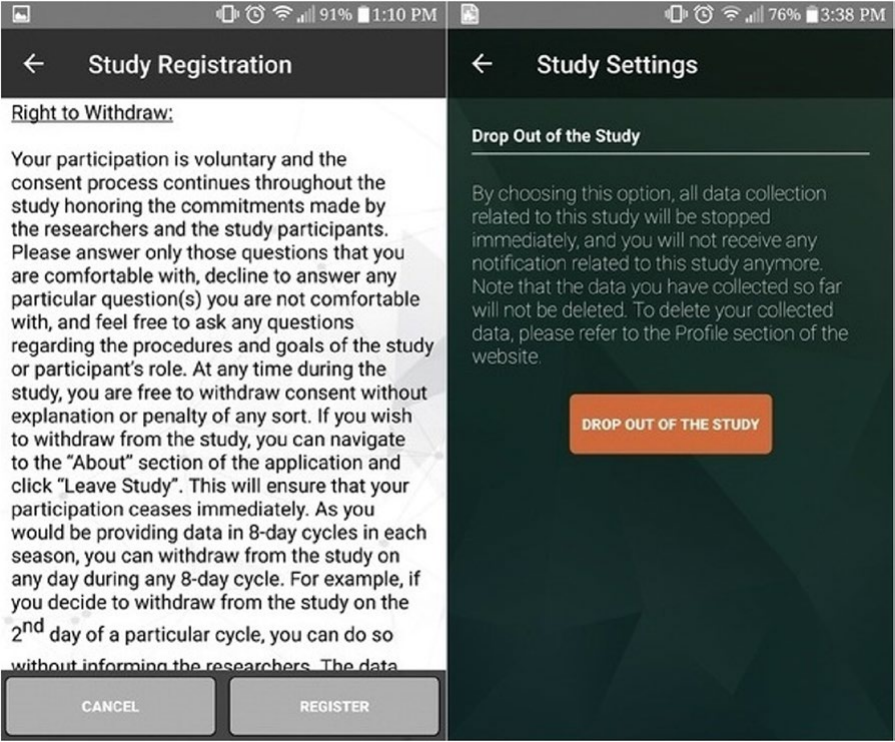
Study Dropout Option in the Smartphone App

Additionally, the smartphone app provided a dropout option, where youth and their caregivers were advised that each participant could refuse to participate in the study or withdraw from the study without any penalty at any time during the data collection cycle (Fig. 3). Participants were provided clear instructions on how to withdraw from the study within the app and these instructions were available to them at all times, via the app [17].

### Data security and anonymity

To ensure data security and privacy of participants, data were anonymized and encrypted before being uploaded to the secure server [20]. The study app that participants downloaded onto their phone did not monitor personal activity, such as texting or internet searching, and was unable to access any personal information on smartphones such as the camera roll or contact lists [20]. Additionally, participants did have the option to disable data collection of the objective sensors (such as GPS and screen state) at their discretion. Finally, if a participant decided to withdraw from the study, but had already participated to some degree, they could have requested to have their data deleted.

### Measures

All data were collected through a combination of validated self-report surveys on participants’ smartphone devices during the school year in a single 8-day period [12, 21]. Participants completed the 124-item Youth Survey, which included validated self-report surveys that collected data on smartphone use and screen time behaviors, demographic characteristics, and health outcomes such as weight status and self-rated health.

#### Smartphone use

Participants in this study were asked to self-report the amount of time (in minutes) that they spend engaged in smartphone specific screen time activities during the school year, on a typical weekday and weekend day. To accomplish this, the Youth Survey included a modified version of the 9-question Sedentary Behavior Questionnaire (SBQ) used to measure screen time use in general [22]. The SBQ was specifically modified to capture comprehensive smartphone use [17] and included the following behaviors: (1) internet surfing (e.g., Facebook, Snapchat, Instagram, YouTube, Reddit, reading news, etc.), (2) playing video games, and (3) texting.

Using the self-reported data collected, separate smartphone use variables were calculated for weekday and weekend variables for the following behaviors: (1) internet use, (2) gaming, (3) texting, and a sum of the three behaviors to estimate (4) total smartphone screen time. Four additional variables were created to capture weekly smartphone activity. Smartphone use was calculated for a full week using the sum of the weekday variable multiplied by 5 and the weekend day variable multiplied by 2 for the following behaviors: (1) weekly internet use, (2) weekly gaming, (3) weekly texting, and (4) weekly smartphone screen time.

There are currently no public health recommendations for smartphone screen time specifically. However, the general recommendation for youth recreational screen time use is no more than two hours per day [23]. As such, in this study, “high smartphone use” will be defined as “more than two hours” for weekdays and weekend days, and “more than 14 hours” for weekly smartphone use.

#### Weight status

Participants self-reported their height in centimeters and weight in pounds. Height and weight were converted to meters and kilograms respectively so that BMI could be calculated, and weight status could be determined. For the BMI calculations, first, raw BMI was calculated (kg / m / m). Then we used the ‘egen’ function in Stata 15.0 to generate BMI categories. The ‘egen’ function with the zbmicat function was used while specifying the WHO 2007 growth charts and which variables age and sex correspond to so that the BMI classification is age and sex specific, in line with the guidelines. World Health Organization BMI cut-offs [24] were used to interpret weight status categories such as thinness, healthy weight, overweight, and obesity [25–27]. For the purpose of this study, a binomial regression was conducted to analyze overweight/obesity compared to healthy weight.

#### Self-rated health

Previous studies have shown that people’s assessments of their own health is a reliable tool [28, 29] with increasing power and validity over time. For example, Schnittker & Bacak (2014) found that self-rated health surveys had greater strength in prediction from 1980 to 2002. To determine the self-rated health, participants were asked to respond to the following question: “In general, would you say your health is…” Participants were provided choices of “Very good,” “Good,” “Fair,” “Bad,” or “Very Bad.” Responses were amalgamated to represent categories of “Very good/Good” versus “Fair/Bad/Very bad.” A binomial regression was conducted to analyze those who felt they have good health vs. those who felt they have poor health.

#### Control variables

Each statistical model in this study controlled for gender, school, grade, and ethnicity. As part of the 124-item survey, participants responded to the question “What is your gender?” with three response options: female, male, transgender, other, or prefer to not disclose. As such, gender was a categorical variable in this analysis. The smartphone surveys also asked participants what school they attended (1 of 5 participating schools in Saskatchewan; schools was a categorical variable in this analysis), and what grade they were in (grades 9, 10, 11, or 12; grade was a continuous variable in this analysis). Finally, while the survey allowed for a variety of options to choose ethnicity, for the purpose of this study, given the Canadian context, categories were adapted to represent the Indigenous, Canadian, and other ethnicity populations. Ethnicity was a categorical variable in this analysis.

### Statistical analyses

All analyses were completed using Stata version 15.0 with significance set at *p* < 0.05. Table 1 shows the demographic frequencies calculated for binary and categorical variables and means and standard deviations calculated for continuous variables. Binary regression models were used to assess the association between measures of physical health (weight status and self-rated health) as the dependent variables and smartphone use as the main independent variable. To account for unique smartphone behaviors, separate models were conducted to assess internet, gaming, and texting versus total smartphone use, which was the sum of all three behaviors. Each model controlled for gender school, grade, and ethnicity. The sample size was determined by power calculations that indicated a sample of 306 would be sufficient for *p* < 0.05 with a power of 0.8 and odds ratios above 2.0.

### Data and risk management

To ensure confidentiality, data were encrypted before being stored on the smartphones and streamed to servers when devices established Wi-Fi connection. Any identifiable artefacts (e.g., photos) were removed or de-identified before data analysis. Permissions built into the app are restricted so that the app cannot access personally identifiable information that is present on the smartphones (e.g., contact list or network sites visited). MAC address anonymization was used to protect citizen scientists’ data based on a simple hash algorithm. Risks and privacy management options were made clear to citizen scientists while obtaining informed consent. All citizen scientists had the option to drop out of the study or pause data gathering anytime they wished via the app. Moreover, they also had the option in the settings of the app to upload data only when they had WI-FI access and/or when they were charging their phones [17]. Clear instructions were provided regarding study withdrawal within the app.

## Results

A total of 808 youth, aged 13–21 years, were recruited for this study. Of these, 436 participants provided data on smartphone use were included in this study. Table 1 provides characteristics of the sample, where 55.7% identified as female, 38.5% as male, and 5.7% as transgender, other, or preferred not to disclose their gender. Categories for ethnicity were adapted to represent the Indigenous (5%), Canadian (39.8%), and other (55.2%) populations. Youth were recruited from grades 9 (29.7%), 10 (20.4%), 11 (14.5%), and 12 (35.4%) and the average age was 16 years old.

**Table 1:** Summary characteristics of smart youth (*N* = 436)

	Proportion (in percent)	Frequency (n)
Grade		
9	29.7	125
10	20.4	86
11	14.5	61
12	35.4	149
School		
1	25.3	110
2	17.1	74
3	11.5	50
4	18.0	78
5	28.1	122
Ethnicity		
Indigenous	5.0	21
Canadian	39.8	166
Other	55.2	230
Gender		
Female	55.7	233
Male	38.5	161
Transgender/other/did not disclose	5.7	24
	**Mean**	**SD**
Age	16.0	1.8

**Fig. 4:**
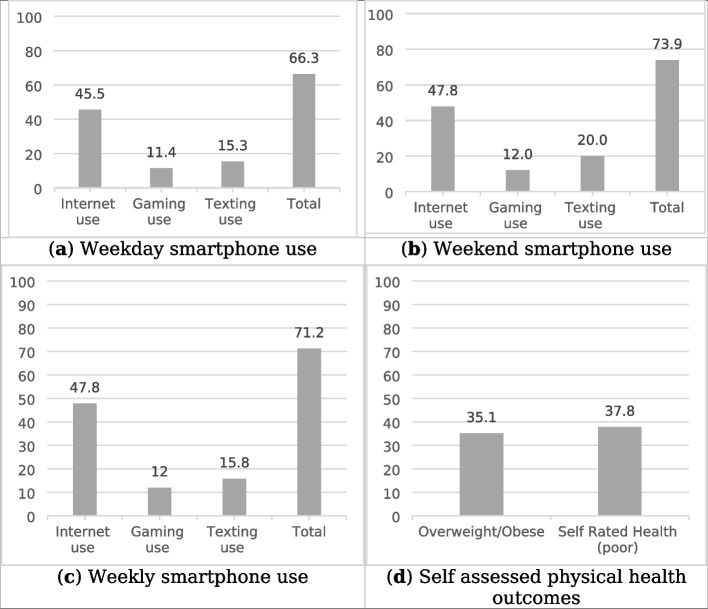
Proportion (in percent) of youth reporting high smartphone use (> 2 h/day), high weight status, and poor self-rated health. **a** High smartphone use on weekdays. **b** High smartphone use on weekends. **c** High smartphone use during a typical week. **d** High weight status and poor self-rated health.

The proportion of youth reporting high smartphone use is represented in Fig. 4a-c, and poor physical health and self-rated health in Fig. 4d. For high smartphone use on a typical week day during the school year (Fig. 4a), 45.5% of participants reported using their smartphone for two hours or more for internet; 11.4% for gaming; 15.3% for texting; and 66.3% for all behaviors combined. On a typical weekend day during the school year (Fig. 4b), 47.8% of participants reported using their smartphone for two hours or more for internet use; 12% for gaming; 20% for texting; and 73.9% for all behaviors combined. Finally, for a full week during the school year (Fig. 4c), 47.8% of participants reported using their smartphone for 14 or more hours of internet; 12.0% gaming; 15.8% texting; and 71.2% on all behaviors combined.

Figure 4d shows the proportion of youth reporting overweight/obese weight status, as well as, poor self-rated health. In terms of weight status, 35.1% of participants would be categorized as overweight or obese. As well, 37.8% of youth in this study rated their general health poorly.

Results from the regression model that assessed the relationship between excessive smartphone use and weight status can be found in Table 2 (Model 1–6). Participants who engaged in excessive gaming screen time during a typical week and weekday were 2.46 and 2.50 times more likely than their peers to fall within an overweight/obese BMI status (typical week: 95% C.I. = 1.19–5.13, results from Model 1; weekend: 95% C.I. = 1.17–5.32, results from model 3), respectively.

**Table 2:** Association between weight status ^a^ (dependent variable) and high smartphone use (independent variables), respectively, among a sample of youth in Canada

	**Weekly Smartphone Use** ^**b**^		**Weekend Day Smartphone Use** ^**c**^		**Weekday Smartphone Use** ^**c**^	
	**OR**	**95% CI** ***p*-value**	**OR**	**95% CI** ***p*-value**	**OR**	**95% CI** ***p*-value**
	**Model 1**		**Model 2**		**Model 3**	
Internet use	1.00	(0.62–1.58) 0.965	0.62*	(0.39–0.98) 0.043	0.91	(0.57–1.45) 0.702
Gaming use	2.46*	(1.19–5.13) 0.016	2.02*	(1.00–4.04) 0.047	2.50*	(1.17–5.32) 0.018
Texting use	1.13	(0.59–2.15) 0.708	1.00	(0.57–1.75) 0.995	1.27	(0.66–2.42) 0.471
	**Model 4**		**Model 5**		**Model 6**	
Total smartphone use	0.74	(0.47–1.17) 0.198	0.65	(0.40–1.04) 0.073	0.87	(0.56–1.34) 0.530

All models controlled for grade, ethnicity, school, and gender

^a^Overweight and obese vs. healthy weight status

^b^Consisted of the sum of 5 weekend and 2 weekday smartphone use. Smartphone use of over 14 h versus less than 14 h (Ref.)

^c^Smartphone use over 2 h versus less than 2 h (Ref.)

* *p *<0.05, ** *p *<0.01, *** *p*< 0.001

Model 1 contained all of: internet use, gaming use, and texting use in the same model, as did models 2, and 3. While models 4, 5, and 6 contained a variable that is the sum of all three smartphone behaviours and the control variables only (i.e., no other smartphone use variables were included).

Results from the regression model that assessed the relationship between excessive smartphone use and self-rated health can be found in Table 3 (Model 7–12). Participants who engaged in excessive gaming screen time over a typical weekend day were 2.34 times more likely than their peers to report poor self-rated health (95% C.I. = 1.22–4.61, results of Model 8).

**Table 3:** Association between self-rated health ^a^ (dependent variable) and high smartphone use (independent variables)

	**Weekly Smartphone Use** ^**b**^		**Weekend Day Smartphone Use** ^**c**^		**Weekday Smartphone Use** ^**c**^	
	**OR**	**95% CI** ***p*-value**	**OR**	**95% CI** ***p*-value**	**OR**	**95% CI** ***p*-value**
	**Model 7**		**Model 8**		**Model 9**	
Internet use	0.96	(0.60–1.51) 0.849	0.87	(0.55–1.37) 0.539	0.91	(0.58–1.44) 0.692
Gaming use	1.54	(0.79–3.03) 0.206	2.38*	(1.22–4.61) 0.011	1.64	(0.82–3.27) 0.162
Texting use	1.55	(0.83–2.89) 0.169	1.20	(0.69–2.08) 0.521	1.43	(0.77–2.69) 0.258
	**Model 10**		**Model 11**		**Model 12**	
Total smartphone use	1.20	(0.75–1.92) 0.454	1.57	(0.95–2.58) 0.079	1.47	(0.93–2.32) 0.097

All models controlled for grade, ethnicity, school, and gender

^a^ Fair/Bad/Very Bad vs. very good/good self-rated health

^b^ Consisted of the sum of 5 weekend and 2 weekday smartphone use. Smartphone use of over 14 h versus less than 14 h (Ref.)

^c^ Smartphone use over 2 h versus less than 2 h (Ref.)

* *p *<0.05, ** *p *<0.01, *** *p*< 0.001

Model 7 contained all of: internet use, gaming use, and texting use in the same model, as did models 8, and 9. While models 10, 11, and 12 contained a variable that is the sum of all three smartphone behaviours and the control variables only (i.e., no other smartphone use variables wereincluded).

**Fig. 5:**
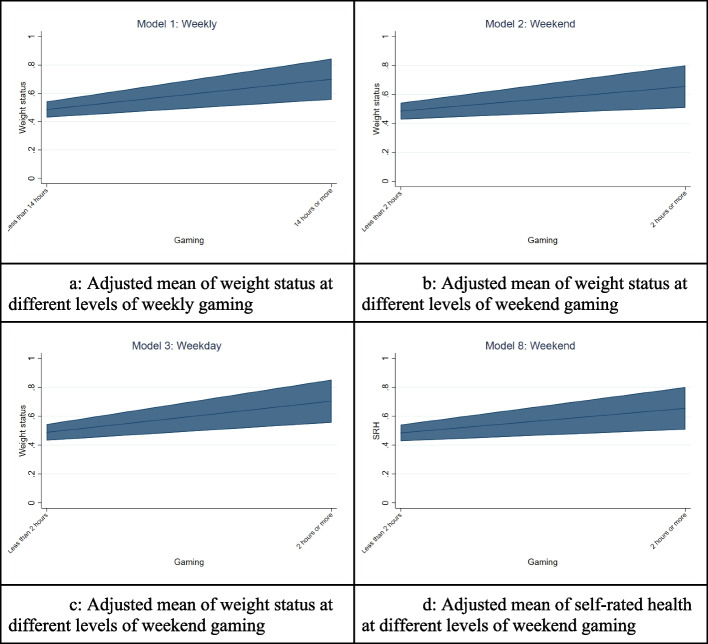
Adjusted mean of weight status (**a**, **b**, **c**) and self-rated health (**d**) at different levels of gaming. All models controlled for internet use, texting use, gender, ethnicity, grade, and school

Figure 5 shows the estimated adjusted mean of weight status and self-rated health across the significant smartphone use behaviours from the regression models. We directed our attention to gaming since it was the significant findings across four models. Figure 5a shows the significant results from Model 1, as gaming increases, so does the adjusted mean of weight status. The findings are also similar across Fig. 5b (significant findings from model 2), Fig. 5c (significant findings from model 3). This trend is also seen in Fig. 5d, as gaming increases, so does the adjusted mean of reporting poor self-rated health (significant findings from model 8).

## Discussion

The objective of this study was to investigate the relationship between smartphone use and physical health outcomes, namely weight status and self-rated health. This research was implemented by using the Smart Platform, which is a citizen science and digital epidemiological initiative for ethical population health surveillance, integrated knowledge translation, and real-time behavioral interventions [17]. The findings from this study build on the current evidence which indicates that those who engage in excessive screen time are at greater risk of poor health outcomes [2, 16, 31]. However, there are several findings in this study that address gaps in the current body of knowledge with respect to what is known about youth and the most commonly used digital devices – smartphones [8, 9].

It is well documented that screen time behaviors, including smartphone use, is strongly associated with poor physical fitness and a higher weight status in the adult population [32–35] and although less evidence is available, these associations are also found with youth as well [15, 36]. Our study also found that youth who engaged in extensive gaming using their smartphones were at a higher risk of living with overweight or obesity. Our study is unique in that our results showed these associations were true for youth who reported 2 h or more of smartphone gaming for both week and weekend days.

However, some research indicates that findings regarding weight status and video gaming among youth remains ambiguous [39]. This may be in part due to emerging popularity of active video gaming which encourages youth to move while participating in screen time [38, 39]. However, a meta-analysis conducted by Bochner, Sorensen, and Belamarich (2015) indicated that even though active screen time behaviors may increase energy expenditure, it is generally not high enough to be associated with lower weight status or weight loss in youth. Our findings add new evidence that excessive gaming using smartphones is associated with a higher risk of living with overweight or obesity among youth. This is an important finding due to the ubiquitous nature of these digital devices and adds to the building body of evidence that there is a need to develop smartphone-specific screen time guidelines for youth [12].

We also found that youth who engaged in excessive smartphone gaming on weekends were at a higher risk to report fair or bad or very bad self-rated health. Self-rated health is a powerful predictor for morbidity and mortality [28] and also a concept commonly used as a health indicator for both eudaimonic wellbeing and quality of life [40–43]. There is an abundance of literature that concludes physical inactivity and high rates of sedentary behavior have negative associations with self-rated health [44–46]. Overall, research suggests that youth who engage in greater than 2 h per day of screen time activities are at risk for poor self-rated health [47]. For example, research focused on traditional types of screen time use such as television viewing and computer/video gaming, showed that higher television viewing time, not computer/video gaming screen time was associated with poor self-rated health, but only for males [16]. By contrast, higher television viewing time and computer/video gaming screen time had other negative health impacts for females; namely, with social friendships [16]. However, such studies consider screen time activity using computer, video games, internet surfing, and television, whereas this study addresses a gap in our understanding of screen time and provides evidence specifically using smartphone devices with the youth population.

Overall, this study is unique because it focuses specifically on youth citizen science, as part of the Smart Platform [17]. Citizen science is a participatory approach to conduct research, where researchers collaborate with participants starting from data collection through to knowledge translation [17, 20, 48]. Using reliable and accessible digital tools and technologies, such as smartphones, can enhance citizen science approaches [17, 20]. The data collected for this study leveraged the market buy-in of citizen-owned smartphones to better understand youth screen time behaviours. Furthermore, all participants completed the 124-item Youth Survey which included a modified version of the 9-question SBQ used to measure sedentary behavior and screen time accumulation [22]. The purpose of adapting this survey was to capture complex screen time-based behaviors (internet surfing, gaming, texting) on a variety of digital devices (computer, laptop, tablet, smartphone) [17].

While screen time guidelines remain focused on limiting actual time spent on screens overall [49] there are health organizations that provide suggestions, particularly to parents and guardians, about considering not only the amount of time on screens but rather the quality of the content that youth are engaged in [50]. This study reiterates the importance of building healthy screen habits by showing that there are variations in health outcomes dependent on the type of screen time behavior. For example, we studied internet, gaming, and texting behaviors specifically on smartphones, where gaming activity was the only behavior associated with negative health outcomes. Future research should focus on a multi directional relationship between health outcomes and specific gaming activities on smartphones by segregating active and passive gaming [38, 39].

### Strengths and limitations

The key strength of this study is that we utilized youth citizens’ own smartphones, to ethically and efficiently collect data about health outcomes and screen time behaviors. Additionally, this study adapted validated self-reported surveys to further investigate behaviors specific to smartphone devices, by not only focusing on overall smartphone use, but also individual behaviors (gaming, texting etc.). However, as with all research that relies on participant recall of either a validated survey that was modified (screen time), or a single-time question (self-rated health), the validity of the measures have limitations. For example, this study asked youth to self-report their weight and height. Although objective measures by a trained professional are recommended, given the digital nature of the survey, objective measures would not be feasible. Additionally, though associated with weight status, our analysis did not include dietary intake as it was not collected as part of the survey. Physical activity information was collected; however, we did not find significant differences in physical activity across the BMI groups, thus we did not include it in the analyses for the parsimony of the models. It is also important to note that this was an exploratory investigation between smartphone use and physical health, which did not delve into deeper pathways such as gender differences in smartphone use. Future studies should conduct gender-stratified analyses between smartphone use, weight status, and self-reported health.

Finally, as this is a cross-sectional study conducted in a single jurisdiction, future longitudinal research should try to replicate the approach employed in this study using the Smart Framework [20] in multiple jurisdictions to expand generalizability and representativeness.

## Conclusion

The results from this study indicate that youth who engage in greater than 2 hours/day of screen time on their smartphones are associated with almost 3 times higher weight status, and report poor general health than their peers who spend less time on their smartphones. This study investigates multiple smartphone screen time behaviors (internet, gaming, and texting) and highlights that different smartphone screen time behaviors have unique associations with health status of youth. Current screen time guidelines do not take into account the nuance of screen time accumulation across different digital devices. In this digital age, with youth consistently reporting significantly higher smartphone use, it is evident that further investigation with more robust study designs is needed to inform smartphone-specific screen time guidelines for youth.

## Data Availability

The study is part of the Smart Platform, a citizen science and mHealth initiative for ethical surveillance, integrated knowledge translation, and policy and real-time interventions. As this study contains sensitive data such as time-stamped location of citizen scientists, data requests should be sent to the University of Regina’s Research Ethics Board at research.ethics@uregina.ca.

## Abbreviations

BMI: Body Mass Index
SBQ: Sedentary Behavior Questionnaire
OR: Odds Ratios
